# Adherence with tobramycin inhaled solution and health care utilization

**DOI:** 10.1186/1471-2466-11-5

**Published:** 2011-01-20

**Authors:** Becky A Briesacher, Alexandra L Quittner, Lisa Saiman, Patricia Sacco, Hassan Fouayzi, Lynne M Quittell

**Affiliations:** 1 University of Massachusetts Medical School and Meyers Primary Care Institute, Worcester, MA, USA; 2 University of Miami, Department of Psychology, Coral Gables, FL, USA; 3 Columbia University, Department of Pediatrics, New York, NY, USA; 4 Novartis Pharmaceuticals Corporation, East Hanover, NJ, USA; 5 Meyers Primary Care Institute, Worcester, MA, USA; 6 Columbia University, Department of Pediatrics, New York, NY, USA

## Abstract

**Background:**

Adherence with tobramycin inhalation solution (TIS) during routine cystic fibrosis (CF) care may differ from recommended guidelines and affect health care utilization.

**Methods:**

We analyzed 2001-2006 healthcare claims data from 45 large employers. Study subjects had diagnoses of CF and at least 1 prescription for TIS. We measured adherence as the number of TIS therapy cycles completed during the year and categorized overall adherence as: low ≤ 2 cycles, medium >2 to <4 cycles, and high ≥ 4 cycles per year. Interquartile ranges (IQR) were created for health care utilization and logistic regression analysis of hospitalization risk was conducted by TIS adherence categories.

**Results:**

Among 804 individuals identified with CF and a prescription for TIS, only 7% (n = 54) received ≥ 4 cycles of TIS per year. High adherence with TIS was associated with a decreased risk of hospitalization when compared to individuals receiving ≤ 2 cycles (adjusted odds ratio 0.40; 95% confidence interval 0.19-0.84). High adherence with TIS was also associated with lower outpatient service costs (IQR: $2,159-$8444 vs. $2,410-$14,423) and higher outpatient prescription drug costs (IQR: $35,125-$60,969 vs. $10,353-$46,768).

**Conclusions:**

Use of TIS did not reflect recommended guidelines and may impact other health care utilization.

## Background

Over the past 20 years, median life expectancy for patients with cystic fibrosis (CF) has increased; recent data from the Cystic Fibrosis Foundation Patient Registry demonstrated a median survival of 37.4 years among patients in the United States (US)[1]. These improvements in survival are associated with earlier diagnosis, more active treatment of pulmonary and digestive complications, and a growing number of effective therapies [2]. One of the consequences of these medical advances is the increased duration and complexity of daily treatments for CF [3]. As research in other chronic illnesses has shown, both time and complexity of daily regimen are associated with lower rates of adherence [4], and these observations may also be true for the CF population[5].

In CF, bacterial inflammation and infections of the lungs are common, particularly those caused by the pathogen *Pseudomonas aeruginosa *(*P. aeruginosa*). By the age of 18, approximately 80% of individuals with CF test positive for *P. aeruginosa*[1,6]. Individuals with CF and *P. aeruginosa *infections have twice the risk of hospitalization for acute respiratory exacerbations and twice the risk of death as those without *P.aeruginosa *infections[7,8]. The aminogylycosdies tobramycin, gentaicin and colistin are active against P. aeruginosa and these drugs are administered by inhalation, although only tobramycin inhalation solution (TIS) is approved for inhalation. The *Cystic Fibrosis Pulmonary Guidelines: Chronic Medications for Maintenance of Lung Health *recommend the chronic use of TIS if *P. aeruginosa *is persistently present in airway cultures, regardless of the severity of the disease, although the recommendation is strongest in the case of moderate to severe disease [9].

Reported rates of prescribing TIS to eligible patients are highly variable, ranging from 47-90% [3,10,11]. Estimates from the CF Foundation patient registry suggest that nearly 58% of individuals with CF and *P. aeruginosa *have had TIS prescribed at least once, although there are no estimates of chronic use[9]. Other estimates of TIS use have been limited by the lack of data beyond initial receipt of the medication and the use of self-reported questionnaires, which can influence adherence estimates[3,4,12,13].

In the U.S., the majority of patients with CF receive their health care through a network of over 120 CF care centers and affiliated programs that are located nationwide. CF centers provide multi-disciplinary care teams of physicians, nurses, social workers, and respiratory therapists. Approximately 60% of CF patients have their health care services and products paid for by employer-based health insurance that is offered to employees and their dependents as part of the employment benefit package[1]. Detailed insurance claims are generated for every prescription drug, hospitalization, and physician service received by patients with CF and thus claim-based databases are one of the most comprehensive sources for assessing the overall health care utilization of this patient population in the U.S. [14].

The objectives of this study were to: 1) measure adherence with TIS as documented in prescription claims data and 2) assess the relationship between use of TIS and health care utilization, particularly hospitalizations.

## Methods

### Data

This study used a research database of health care claims, the 2001-2006 Medstat MarketScan Commercial Claims and Encounters and Medicare Supplemental Databases (Ann Arbor, MI). This national database contains over 500 million claim records per year from individuals with private health care insurance in the United States. Scientific studies based on this data source have been reported in more than 75 peer-reviewed articles [15]. The data come from approximately 45 large employers who self-insure their employees and dependents. Typically, large corporations or government entities use self-insurance as a way to manage their risk pool of sick and healthy employees rather than hire an insurance company. Self-insured employers have detailed data on the health care utilization of their employees and dependents since medical care providers must submit claims for reimbursement for all services rendered.

The MarketScan database offers advantages over raw administrative claims because data files undergo validity and editing procedures to ensure high quality and consistency in fields across years [16]. The data are evaluated against population norms, previous year summaries, and validated data subsets. Outliers are flagged and reviewed for coding or processing errors. Encounter data describe the individuals who are covered within the database and are audited at the health plan level and plans submitting incomplete data are excluded. Diagnostic and procedural codes are compared against validity algorithms and set to missing values if inconsistent. The encounter data include age, sex, geographic residence, and eligibility information. The prescription claims include the national drug codes, date of purchase, quantity, days' supply, and expenditure information. The medical claims contain payment information, diagnoses, procedure codes, and type of provider. For this analysis, we pooled annual files to create a dataset of approximately 12 million people.

### Sample

We identified the study sample as individuals who had at least 2 inpatient or outpatient claims with a diagnosis of CF using an International Classification of Disease-9^th ^Revision-Clinical Modification (ICD-9-CM) code of 277.0x and were continuously enrolled in health insurance with drug coverage for at least 1 year (n = 2,515). Using at least 2 claims for the diagnosis of CF has been shown to increase specificity for CF cases by increasing the probability of identifying true cases in administrative datasets, although sensitivity is generally low [17]. Individuals were excluded if they had not used TIS as described below (n = 1,691) or had undergone lung transplantation (n = 20).

### Measure of TIS utilization

We developed a new measure of adherence with TIS to reflect the unique administration of this therapy as a cycle of 28 days on therapy and 28 days off therapy. Adherence was calculated as the sum of the days' supply dispensed during the year divided by 56. Overall adherence categories were defined as: low utilization ≤ 2 cycles, medium utilization > 2 to < 4 cycles, and high utilization ≥ 4 cycles. These categories were defined with clinician input and review of TIS utilization data in the hypothesis generating phase of this study. Adherence, as defined by these annual utilization thresholds, was measured only during the first year of observation, and did not include extemporaneously compounded aerosolized tobramycin.

### Measures of health care utilization

Health care utilization was extracted from all-cause medical claim encounters. Hospitalization was determined by any admission to an inpatient care setting. Total costs were summed over the year and categorized into two main settings of care (i.e., inpatient care and outpatient care), and prescription drugs. We inflated the health care cost values to the year 2006 using factors from the US Bureau of Labor Statistics current data on the consumer price index [18].

### Demographic and Health Measures

We evaluated demographic characteristics, including age, gender, and the 4 U.S. geographic regions of residence (i.e., Northeast, Midwest, South, and West), and type of health insurance plan (i.e., comprehensive, health maintenance organization, preferred provider organization, and other). In addition, we examined the claims data of each individual for primary and secondary diagnoses indicating selected comorbidities. A total burden of comorbidity score was calculated using the Diagnostic Cost Group Hierarchical Condition Category (DCG/HCC) classification system (DxCG, Boston, MA) [19]. The DCG/HCC model produces a risk score for each person based on the presence of 189 medical conditions in the diagnosis fields of claims records. The model uses diagnoses from all sites of service and imposes hierarchies on the resulting condition categories prior to calculating risk scores. The hierarchies identify the most costly manifestation of each distinct disease and decrease sensitivity of the DCG/HCC models to coding idiosyncrasies. For ease of interpretation, we normalized the individual's scores by the population average score, and categorized the study population into two groups, those with less than or average (for the general population) comorbidity risk and those with higher than average risk. In the general population, the DCG/HCC has shown good predictive validity for death, hospitalization, and health care expenditures [20]. In addition, we searched for two CF-related diagnoses, not included in the comorbidity score, *P. aeruginosa *(ICD-9 codes 277.02, 041.7) and Failure to Thrive/Growth Failure (ICD-9 codes 783.41, 783.43).

### Data Analysis

For the statistical analyses, we calculated means, frequency distributions and 95% confidence intervals, and tested group differences using chi-squares and t-tests at the p < 0.05 level. We reported medians and interquartile ranges for all expenditures and used logistic regressions to estimate the probability of hospitalization.

All statistical analyses were carried out with the SAS 9.1.3 (SAS Institute, Inc., Cary, North Carolina). Additionally, this study received an exemption waiver from the University of Massachusetts's Institutional Review Board for the use of previously collected and de-identified data.

## Results

The characteristics of the 804 individuals identified with CF who were receiving TIS therapy are shown in Table 1. About 43% were adults 18 years of age or older, 53% were male, and 66% lived in the South or Midwest region of the U.S. Approximately 12% had diabetes mellitus (data not shown) and nearly 35% had a higher than average comorbidity score. Overall, 56.1% of the sample had a diagnosis of *P. aeruginosa*.

**Table 1:** Characteristics of cystic fibrosis study population receiving tobramycin inhalation solution, N = 804.

Demographic characteristics	%*
**Age, years**	
<6**	13.2
6-10	12.2
11-17	31.5
18-25	22.0
≥26	21.1
	
**Gender**	
Male	52.7
Female	47.3
	
**Comorbidity score**	
≤Average	65.3
>Average	34.7
	
**Geographic region**	
Northeast	12.3
Midwest	27.0
South	39.1
West	21.4
	
**Health Plan Type**	
Comprehensive	7.6
HMO	23.0
Preferred Provider Organization	49.0
Other Plans	20.4
	
**Selected comorbidity**	
*Pseudomonas aeruginosa*	56.1
Failure to thrive/Growth failure	4.4

*percentages may not add up to 100% due to rounding

**< 6 years of age further divided; 3.9% of patients were < 2 years of age and 9.3% were 2-5 years of age.

Chronic us of TIS was low in subjects with *P. aeruginosa *as only 6% were dispensed ≥ 4 cycles per year as shown in Table 2. TIS usage in the three utilization strata was similar for subjects with and without the diagnosis of *P. aeruginosa*.

**Table 2:** Overall adherence with tobramycin inhalation solution by *P. aeruginosa *infection.

	Low utilization ≤2 cycles (n = 570)	Medium utilization >2 to <4 cycles (n = 180)	High utilization ≥4 cycles (n = 54)
All (N = 804)	71%	22%	7%
With *P. aeruginosa *(n = 452)	72%	22%	6%
Without *P. aeruginosa *(n = 352)	70%	23%	7%

In comparison to subjects with high utilization of TIS, those using fewer than 4 cycles a year were more likely to be hospitalized as shown in Table 3. However, among those hospitalized, inpatient costs were similar between the three utilization strata. Median outpatient costs (excluding drug costs) were lower among high TIS users compared to those using fewer cycles. However, median outpatient prescription drug costs were higher among high users. TIS represented between 29% and 45% of total prescription drug costs.

**Table 3:** Health care utilization by overall adherence with tobramycin inhalation solution, N = 804.

	Low utilization ≤2 cycles (n = 570)	Medium utilization >2 to <4 cycles (n = 180)	High utilization ≥4 cycles (n = 54)
Patients hospitalized (%)	42.9%	40.6%	25.9%
Median inpatient costs, IQR	$23,619	$16,814	$20,610
	$11,566-55,446	$9,527-41,021	$8,076-64,799
			
Median outpatient costs, (excludes drug costs) IQR	$6,317	$5,463	$4,033
	$2,564-14,423	$2,410-13,653	$2,159-8,444
			
Median outpatient prescription drug costs, IQR	$17,850	$35,892	$46,708
	$10,353-27,260	$27,482-46,768	$35,125-60,969
			
% of outpatient prescription drug costs for TIS, median	29%	41%	45%

IQR = interquartile range; TIS = tobramycin inhalation solution

High use of TIS was associated with a decreased risk of hospitalization relative to low use (Adjusted odds ratio (AOR) 0.40; 95% CI 0.19-0.84) as shown in Table 4. In contrast, a higher than average comorbidity risk (AOR 7.53; 95% CI 5.20-10.90), a coded diagnosis of *P. aeruginosa (*AOR 3.0; 95% CI 2.13-4.32), and a coded diagnosis of failure to thrive/growth failure (AOR 2.8; 95% CI 1.09-7.14) were all independently associated with an increased risk of hospitalization. Age and gender were not found to be predictive of hospitalization in this analysis.

**Table 4:** Logistic regression of probability of hospitalization of cystic fibrosis patients, N = 804.

	AOR*	95% CI
**Overall adherence with TIS**		
Low (reference)	1.0	
Medium	0.94	0.62-1.41
High	0.40	0.19-0.84
**Age (in years)**		
<6 (reference)	1.0	
6-10	1.76	0.89-3.47
11-17	1.52	0.86-2.69
18-25	1.48	0.80-2.73
≥26	0.73	0.38-1.39
**Gender**		
Male (reference)	1.0	
Female	1.04	0.74-1.44
**Comorbidity score**		
≤Average (reference)	1.0	
>Average	7.53**	5.20-10.90
**Selected comorbidity**		
*Pseudomonas aeruginosa*	3.0**	2.13-4.32
Failure to thrive/growth failure	2.8**	1.09-7.14

*Adjusted for variables in the table and health plan type and geographic residence; **p < 0.05

AOR = adjusted odds ratio; CI = confidence interval; TIS = tobramycin

## Discussion

To our knowledge, this is one of the first studies to assess the link between TIS use in CF patients and health care utilization as captured in health claims data. In this study of 804 individuals with CF, 56% were identified as having *P. aeruginosa*, although only 6% of those with *P. aeruginosa *were dispensed at least 4 cycles during the year of observation. These findings are similar to those found in a previous study of 357 individuals with CF and *P. aeruginosa*, where only 4% were utilizing chronic intermittent TIS according to guidelines [21], although there was overlap in the study samples.

Rates of aerosolized antibiotic use in CF patients have been previously reported. In the CFF Patient Registry, the national average rate of reported use (based on provider prescribing) was 60.8% (range 17.2-94.7%) in 2001 and 61.8% (range 0-100%) in 2006 [22]. However, these data were not analyzed by *P. aeruginosa *status. Analysis of the Epidemiologic Study of CF database showed that 73.1% of patients with *P. aeruginosa *and 33.8% of patients without *P. aeruginosa *used inhaled tobramycin from 2003-2005 and that 49.7% vs. 15.9% of all patients received inhaled tobramycin for chronic vs. exacerbation use, respectively [23]. Neither of these previous analyses assessed dispensing or refill history or if inhaled tobramycin was used according to practice guidelines, and both assessed all formulations of aerosolized tobramycin.

In the pivotal clinical trial of TIS, Ramsey et al. noted that subjects in the tobramycin group were 26% less likely to be hospitalized and 36% less likely to be treated with intravenous antibiotics than subjects in the placebo group [24]. In the current study, CF subjects with high utilization (≥ 4 cycles per year) had fewer hospitalizations than those subjects with medium and low utilization; the risk of hospitalization among those with high utilization was decreased by 60% compared with those taking 2 or fewer cycles per year. Thus, our findings are consistent with the clinical trial results, and suggest that TIS use in routine clinical care and not within a clinical trial may also be associated with a reduction in hospitalizations. This finding deserves further study as the sample size of CF patients with high utilization was modest (n = 54) and many factors besides TIS adherence contribute to hospitalization risk.

We noted that while median outpatient costs, excluding drug costs, were lower for high users, median outpatient drug costs were higher. Previous studies have sought to examine whether the cost of TIS can be partially offset by reductions in the use of other health care resources. LeLorier et al. and Iles et al. have shown that the use of TIS could potentially reduce the use of health care services, particularly hospitalization and intravenous antibiotic use (29, 30). Furthermore, while not measured by studies to date, decreased hospitalization is likely to result in additional cost saving resulting from fewer disruptions of work and school as well as improved quality of life for patients and caregivers

Poor adherence to TIS, as measured in this study may be due to several factors. Patients may not have a complete understanding of TIS and its risks and benefits and they also have several other medications and treatments to complete each day. In addition to the treatment burden, use of TIS may be limited because of barriers such as taste, portability of the treatment or inability to pay the coinsurance (31). Although TIS is recommended for patients with *P. aeruginosa *infection, prescribing patterns are not consistent nationally, and TIS is likely increasingly being used both as part of early eradication protocols and to treat CF pulmonary exacerbations. Thus, low utilization or fewer annual cycles may reflect these practice patterns.

There are some limitations to this study. This study used pharmacy claims records to measure adherence, and thus our analysis assessed rates of medication acquisition rather than medication exposure. However, research has demonstrated predictive validity for measuring the cumulative exposure of medications with acquisition data[25]. It should be noted, though, that studies using electronic data capturing methods have found a range of adherence rates with some reporting higher levels of TIS adherence in selected populations[26]. Second, *P. aeruginosa *infection and the rate of failure to thrive may be under-coded due to lack of financial incentive to add these diagnoses to billing records. Additionally, the DCG/HCC model has not, to our knowledge, been previously applied to a CF population. Thus, its performance as validated in general populations may vary from what was observed in our study population. However, there is value to applying a standard risk-adjustment methodology to minimize the differences in comorbidity levels observed in the sample. In this dataset we do not have an objective measure of disease severity, such as forced expiratory volume in one second (FEV_1_), to correlate lung health with TIS use. Further, it should be noted that our measure of adherence does not assess all aspects of physician prescribing patterns (e.g. use of generic tobramycin). Also, this study population may not be generalizable to the American CF population; although the proportion of males and adult patients is comparable to that reported to the Patient Registry in 2007, the distribution of geographic regions is not comparable to Registry data (1). Fewer patients from the Northeastern United States were identified for inclusion in the current study, although the impact of geographic region on prescribing patterns for TIS and access to care are unknown. This dataset reflects patients with private health care insurance and findings may not be generalizable to patients on public assistance. Finally, this analysis was limited to one year of observation and did not assess utilization patterns after that period.

The value of a dataset derived from administrative claims data may be unfamiliar to CF clinicians, however, such data are appropriate for assessing many facets of quality of care. One particular strength of these data is the ability to capture medication adherence as measured through prescription dispensing; in this study, we found that persistent use of TIS was quite low and deserves further investigation. We are currently conducting a randomized controlled trial across 19 CF Care Centers (iCARE: I Change Adherence and Raise Expectations Study, NCT01232478) to identify effective strategies that improve adherence in adolescents with CF.

## Conclusions

High utilization of TIS was infrequent yet associated with a decreased risk of hospitalization. However, the low utilization of TIS in subjects with *P. aeruginosa *noted in this study is of concern. These data suggest that CF patients may not be using TIS as recommended by the CF Pulmonary Guidelines. Future studies to address ways to improve adherence are warranted to ensure patients can benefit from their chronic treatments.

## Abbreviations

AOR: adjusted odds ratio; CF: cystic fibrosis; DCG/HCC: diagnostic cost group hierarchical condition category; FEV_1_: forced expiratory volume in 1 second; ICD-9-CM: international classification of disease-9^th ^revision-clinical modification; IQR: interquartile range; *P. aeruginosa*; *Pseudomonas aeruginosa*; TIS: tobramycin inhalation solution.

## Competing interests

Dr. Briesacher, Mr. Fouayzi, Dr. Quittner and Dr. Quittel serve as consultants for Novartis Pharmaceuticals Corporation. Dr. Saiman has served on advisory boards for Novartis. Ms. Sacco is an employee of Novartis Pharmaceuticals Corporation.

## Authors' contributions

BB obtained funding for the study, provided study supervision, and drafted the manuscript. BB had full access to all of the data in the study and takes responsibility for the integrity of the data and the accuracy of the data analysis. BB, AQ and PS conceived the study and participated in the study design. BB and PS provided administrative, technical, or material support, and participated in the acquisition of data. BB and HF participated in the statistical analysis. All authors participated in the analysis and interpretation of the data, provided critical revision of the manuscript for important intellectual content, and approved the final manuscript.

## Pre-publication history

The pre-publication history for this paper can be accessed here:

http://www.biomedcentral.com/1471-2466/11/5/prepub
